# Identification and validation of a novel tumor driver gene signature for diagnosis and prognosis of head and neck squamous cell carcinoma

**DOI:** 10.3389/fmolb.2022.912620

**Published:** 2022-10-20

**Authors:** Shixian Liu, Weiwei Liu, Zhao Ding, Xue Yang, Yuan Jiang, Yu Wu, Yehai Liu, Jing Wu

**Affiliations:** ^1^ Department of Otolaryngology-Head & Neck Surgery, The First Affiliated Hospital of Anhui Medical University, Hefei, China; ^2^ Anhui Medical University, Hefei, China; ^3^ Graduate School of Anhui Medical University, Hefei, China

**Keywords:** signature, HNSCC, WGCNA, prognosis, diagnosis

## Abstract

**Objective:** Head and neck squamous cell carcinoma (HNSCC) is a common heterogeneous cancer with complex carcinogenic factors. However, the current TNM staging criteria to judge its severity to formulate treatment plans and evaluate the prognosis are particularly weak. Therefore, a robust diagnostic model capable of accurately diagnosing and predicting HNSCC should be established.

**Methods:** Gene expression and clinical data were retrieved from The Cancer Genome Atlas and Gene Expression Omnibus databases. Key prognostic genes associated with HNSCC were screened with the weighted gene co-expression network analysis and least absolute shrinkage and selection operator (LASSO) Cox regression model analysis. We used the timeROC and survival R packages to conduct time-dependent receiver operating characteristic curve analyses and calculated the area under the curve at different time points of model prediction. Patients in the training and validation groups were divided into high- and low-risk subgroups, and Kaplan-Meier (K-M) survival curves were plotted for all subgroups. Subsequently, LASSO and support vector machine algorithms were used to screen genes to construct diagnostic model. Furthermore, we used the Wilcoxon signed-rank test to compare the half-maximal inhibitory concentrations of common chemotherapy drugs among patients in different risk groups. Finally, the expression levels of eight genes were measured using quantitative real-time polymerase chain reaction and immunohistochemistry.

**Results:** Ten genes (*SSB*, *PFKP*, *NAT10*, *PCDH9*, *SHANK2*, *PAX8*, *CELSR3*, *DCLRE1C*, *MAP2K7*, and *ODF4*) with prognostic potential were identified, and a risk score was derived accordingly. Patients were divided into high- and low-risk groups based on the median risk score. The K-M survival curves confirmed that patients with high scores had significantly worse overall survival. Receiver operating characteristic curves proved that the prognostic signature had good sensitivity and specificity for predicting the prognosis of patients with HNSCC. Univariate and multivariate Cox regression analyses confirmed that the gene signature was an independent prognostic risk factor for HNSCC. Diagnostic model was built by identifying eight genes (*SSB*, *PFKP*, *NAT10*, *PCDH9*, *CELSR3*, *DCLRE1C*, *MAP2K7*, and *ODF4*). The high-risk group showed higher sensitivity to various common chemotherapeutic drugs. *DCLRE1C* expression was higher in normal tissues than in HNSCC tissues.

**Conclusion:** Our study identified the important role of tumor-driver genes in HNSCC and their potential clinical diagnostic and prognostic values to facilitate individualized management of patients with HNSCC.

## Introduction

Head and neck squamous cell carcinoma (HNSCC) is a heterogeneous group of malignancies that can originate in the tongue, mouth, paranasal sinuses, nasopharynx, oropharynx, hypopharynx, or larynx. Globally, it is the sixth most common cancer type and eighth most common cause of cancer-related deaths (Bray et al., 2018). There are more than 650,000 newly diagnosed cases and 330,000 deaths (Tumban, 2019). The following carcinogenic factors may lead to the occurrence and development of HNSCC: exposure of the upper gastrointestinal mucosa to carcinogens, such as tobacco and alcohol; human papillomavirus infection; and less commonly, Epstein–Barr virus infection (Rickinson, 2014; Diez-Fraile et al., 2020).

The current standard treatment for HNSCC is surgery with or without chemotherapy and/or radiotherapy (Sacconi et al., 2020). Approximately two-thirds of patients with locally or regionally advanced disease adopt a certain combination of these three treatments (Cognetti et al., 2008; Rosenberg and Vokes, 2021). Although surgical methods are constantly being updated and new therapeutic approaches are being developed, the survival rate of patients with HNSCC has not improved significantly. Currently, the standard method for clinicians to judge the severity of HNSCC is TNM staging (Syed et al., 2020). However, the TNM stage considers only the anatomical factors of the tumor; therefore, using it to develop treatment plans for patients and assess the prognosis is particularly inefficient. Coupled with the diverse etiology and high degree of heterogeneity of HNSCC, accurate prediction of patient prognosis poses a great challenge. Therefore, robust prognostic models should be developed.

Some gene mutations induce changes in gene information or abnormal expression of their products, which can transform normal cells to tumor cells with malignant biological behaviors (Ma et al., 2021). These mutant genes are often referred to as tumor-driver genes. They promote the development and progression of tumors and pose a threat to human health (Steeg, 2006; Zhu et al., 2013). Models based on tumor-driver gene sets that can predict the prognosis of patients with HNSCC are lacking.

With the rapid growth and development of next-generation sequencing, bioinformatics analysis has been widely used and adopted for microarray platforms and data to further explore the underlying genetic and molecular mechanisms of diseases and detect specific biomarkers of disease (Morganti et al., 2019). Weighted gene co-expression network analysis (WGCNA) is a popular algorithm that enables highly correlated genes to be grouped into the same module, with the advantage of being able to link, including but not limited to, clinicopathological parameters (Zhang and Horvath, 2005; Emilsson et al., 2008). In this study, we used WGCNA to identify tumor-driver genes that are strongly associated with HNSCC. We then screened the training dataset (The Cancer Genome Atlas [TCGA]) for 28 tumor-driver genes that were significantly related to the prognosis and established a prognostic signature using least absolute shrinkage and selection operator (LASSO) and multivariate Cox regression analysis. In addition, we reevaluated the model’s performance using a different independent dataset, i.e., *GSE41613*. Accordingly, we constructed a robust diagnostic model capable of accurately diagnosing HNSCC. In addition, our prognostic model can predict the sensitivity of patients with HNSCC to chemotherapeutic drugs.

## Materials and methods

### Data collection

We obtained expression data from TCGA public database for 546 patients with HNSCC (44 normal and 502 tumor tissues) and follow-up data for each patient (Table 1). The expression and clinical data for patients in the *GSE41613* (97 HNSCC tissues), *GSE127165* (57 laryngeal squamous cell carcinomas and paired adjacent normal tissues), and *GSE37991* (40 paired HNSCC and adjacent carcinomas) datasets were downloaded from the Gene Expression Omnibus (GEO). We downloaded 2,372 tumor-driver genes from an online website (http://ncg.kcl.ac.uk/cancer_genes.php). Data were obtained from databases that were freely available to all and, therefore, did not require ethics committee approval.

**TABLE 1:** Clinical data of patients in the TCGA and the GEO validation cohort.

Variables	Subgroups	TCGA	Variables	Subgroups	GEO
		(N = 502)			(N = 97)
Age	< 60	221	Age	< 60	50
	> = 60	280		> = 60	47
	NA	1	Gender	Female	31
Gender	Female	134		Male	66
	Male	368	Stage	I-II	41
Stage	I	19		III-IV	56
	II	95			
	III	102			
	IV	272			
	NA	14			
Grade	I	62			
	II	300			
	III	119			
	IV	2			
	NA	19			

### WGCNA construction and HNSCC-related modules

To identify the tumor-driver genes associated with HNSCC, we constructed a co-expression network using the “WGCNA” R package based on the gene expression matrix of the training dataset (Langfelder and Horvath, 2008). First, we filtered genes with a small range of fluctuating expression levels across all samples. We then examined the expression matrix in TCGA dataset and removed the missing values. The cluster analysis was used to identify discrete samples, which were subsequently removed. When the average connectivity was infinitely close to 0 and the scale-free topology fit index (*R* ^2^) was almost 0.9, the β value was chosen as the soft threshold power. Subsequently, we calculated the topological overlap matrix (TOM) and used matrix 1-TOM to identify hierarchically clustered genes and modules. To ensure reliability of the results, the minimum number of genes in each module was set to 25, and the module branch merge cut height was set to 0.25. The first principal element of each gene module was identified as module eigengenes (MEs). The k-ME value was used as a measure of intra-module connectivity, which represents the correlation between the gene expression level and ME. The modules (|r| ≥ 0.4) that were strongly related to the tumor were selected for the next analysis, and the genes (red, blue, and black) in the eligible modules were extracted.

### Construction of risk signature

We first extracted the gene expression matrix from TCGA dataset for the three eligible modules (655 genes in total). A univariate Cox regression analysis was used to screen tumor-driver genes associated with overall survival (OS) in patients with HNSCC in TCGA training dataset (*p* < 0.005). Immediately afterwards, we used the LASSO regression analysis for 10-fold cross-validation, filtering tumor-driver genes that were more strongly correlated to avoid overfitting. Finally, we used a multivariate Cox regression analysis to develop an optimal prognostic risk model based on the Akaike information criterion (AIC = 2297.34). In the previous process, the genes used to construct the model and their corresponding coefficients were obtained, and a formula for calculating the patient’s risk score was derived, as follows: risk score = ˇgene 1* gene 1 expression + ˇgene 2 * gene 2 expression + ····· + ˇgene N * gene N expression. Samples in each TCGA-HNSCC cohort and validation cohort were categorized into low- and high-risk groups, consistent with the median risk score. We used Kaplan–Meier survival curves with a log-rank test to compare the differences in prognosis between the two groups of patients. To assess the predictive power of the prognostic model, we used the Kaplan–Meier receiver operating characteristic (ROC) and Kaplan–Meier “survival” R packages to conduct time-dependent ROC (tROC) analyses and calculated the area under the curve (AUC) at different time points for model prediction.

### Ten-gene risk signature validation

To determine the robustness and generalizability of the developed predictive models, an independent external dataset validation is necessary. Therefore, *GSE41613*, an external dataset, was used to evaluate the model’s performance. Similarly, patients in the validation dataset were divided into high- and low-risk subgroups using the same cutoff values, and Kaplan–Meier survival curves were plotted for both subgroups of patients. A meta-analysis (I^2^ < 50%, fixed-effects model) was performed using TCGA and *GSE41613* datasets to assess the prognostic value of the model in the combined cohort. Furthermore, a stratification analysis was performed to confirm the prognostic importance of the gene signature in all stratified subgroups.

### Alterations and differential expression analysis of ten genes in the TCGA dataset

We used cBioPortal, which is a free web server that allows interactive exploration of cancer genomic data (Cerami et al., 2012), to predict the specific mutational profiles of these ten genes in patients with HNSCC (TCGA, PanCancer Atlas). Most of these ten genes were significantly differentially expressed in normal and tumour tissues, but the differences in expression of SHANK2 and DCLRE1C were not significant (Supplementary Figure S1).

### Establishment and validation of candidate diagnostic biomarkers

To elucidate whether the prognostic model could also serve as a diagnostic model, the genes used to construct the prognostic model were used to screen candidate genes. LASSO is a powerful analysis method that enables both regularization and variable selection to ensure that the model has strong predictive accuracy. In addition, we used another approach (support vector machine recursive feature elimination [SVM-RFE]) to screen the set of diagnostic genes with the highest discriminant ability. Finally, we used the intersecting genes obtained by the two algorithms to construct a diagnostic model. The superior performance of the diagnostic model in a single dataset was not highly convincing; therefore, its performance was re-validated using two other independent datasets (*GSE127165* and *GSE37991*).

### Prediction of chemotherapy drug sensitivity in patients with HNSCC

Since not all patients with HNSCC are sensitive to chemotherapy owing to individual differences, we used the ‘pRRophetic’ R package to predict the drug sensitivity of patients to reduce the financial burden on patients. Commonly used chemotherapeutic drugs in oncology include cisplatin, lapatinib, methotrexate, and docetaxel. The half-maximal inhibitory concentration (IC50) of multiple chemotherapeutic drugs in each patient with HNSCC was calculated using ridge regression, and the accuracy of prediction was assessed using 10-fold cross-validation. We used the Wilcoxon signed-rank test to compare IC50 of common chemotherapy drugs across risk groups.

### Collection of tissue samples

Fresh primary HNSCC samples and corresponding non-tumor tissues were collected immediately after surgical resection at the First Affiliated Hospital of Anhui Medical University. The tissues were transported to the laboratory within 2 h. The collected tissue samples were stored at −80°C until use. Clinical samples were approved by the Research Ethics Review Committee of the First Affiliated Hospital of Anhui Medical University. Written informed consent was obtained from all patients.

### Real-time reverse transcription quantitative polymerase chain reaction (qRT-PCR)

RNA isolation and purification were performed using TRIzol RNA. The Complementary DNA Synthesis SuperMix Kit (ThermoFisher Scientific) was used to synthesize complementary DNA, which was subjected to qRT-PCR using SYBR^®^ Premix Ex TaqTM II (TaKaRa) and the Real-Time System (Lin et al., 2021) (Roche Life Science). The gene expression levels were normalized to GAPDH messenger RNA (mRNA) expression. Supplementary Table S1 shows the primer sequences used.

### Western blotting

Tissues were lysed with the RIPA lysis buffer (Beyotime, Jiangsu, China), separated by polyacrylamide gel electrophoresis using sodium dodecyl sulfate and transferred to polyvinylidene fluoride membranes (Millipore, Billerica, MA, United States ). Closure with 5% skim milk for 1 h was incubated with the primary antibody overnight for primary antibodies (diluted 1:1000) at 4°C overnight. The membranes were then incubated with horseradish peroxidase-conjugated secondary antibody for 1 h at room temperature. Finally, the protein bands were visualized using chemiluminescence (Wan et al., 2021).

### Immunohistochemistry assay

Twelve pairs of tissue samples were approved by the First Affiliated Hospital of the Anhui Medical University. All tissue samples were approved by patients. Tumor tissues were paraffin-sectioned. HNSCC tissue sections were deparaffinized in xylene and dehydrated in ethanol. Antigen retrieval was performed using citrate buffer (pH 6), followed by blocking with bovine serum albumin for 1 h to prevent nonspecific binding of antibodies. After tumor tissues were paraffin embedded, they were incubated with specific primary antibodies (*DCLRE1C*, 1:100, ThermoFisher Scientific) at 4°C overnight. The paraffin sections were then incubated with horseradish peroxidase-conjugated secondary antibodies for 20 min at room temperature. Finally, photographs were acquired using an inverted microscope (Wang et al., 2020). The positive rate was calculated using the immunohistochemistry toolbox plugin in ImageJ software.

## Results

### Tumor-driver genes in the HNSCC-associated modules identified by WGCNA

**FIGURE 1:**
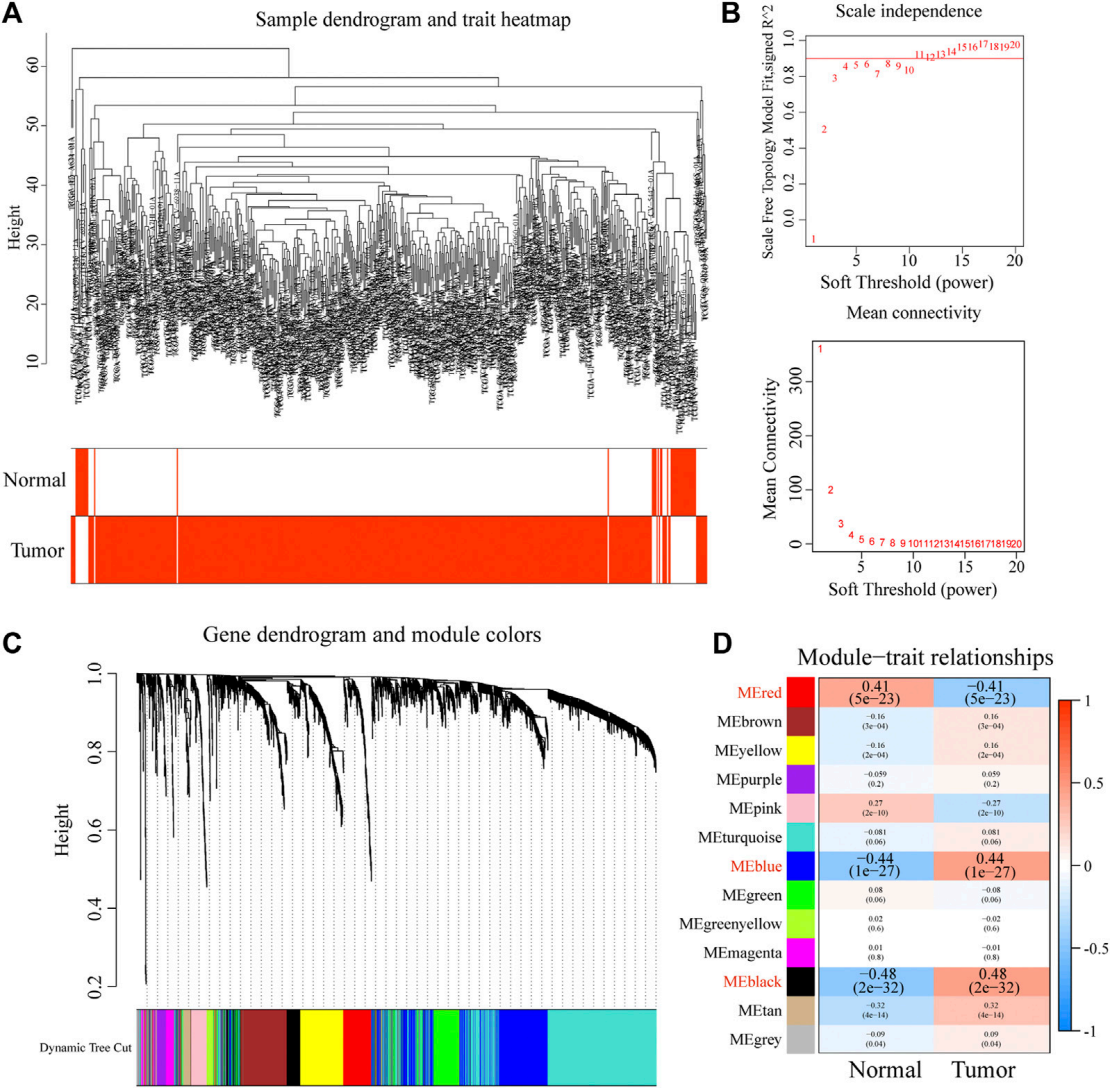
Construction of a co-expression network through WGCNA. **(A)** Clustering dendrogram of HNSCC samples. **(B)** Network topology for different soft-thresholding powers. **(C)** The cluster dendrogram of co-expression network modules is ordered by a hierarchical clustering of genes based on the 1-TOM matrix. **(D)** Module-trait relationships.

To obtain the key modules for HNSCC, we first constructed a co-expression network using 44 normal and 502 HNSCC samples from the training dataset (TCGA cohort; Figure 1A). We then chose β = 4 as the optimal soft threshold, based on which 13 modules were obtained (Figures 1B,C). Furthermore, among these 13 modules, the highest module-trait positive association was found among the red module (*r* = 0.41; *p* = 5e-23), blue module (*r* = 0.44; *p* = 1e-27), and black module (*r* = 0.84; *p* = 2e-32) with HNSCC tissue (Figure 1D). The genes in these modules may play essential biological roles associated with the prognostic signature. Subsequently, 655 genes from the three key modules were selected for further analyses (Supplementary Table S2).

### Establishment of a tumor-driver gene-related signature for the prognosis

**FIGURE 2:**
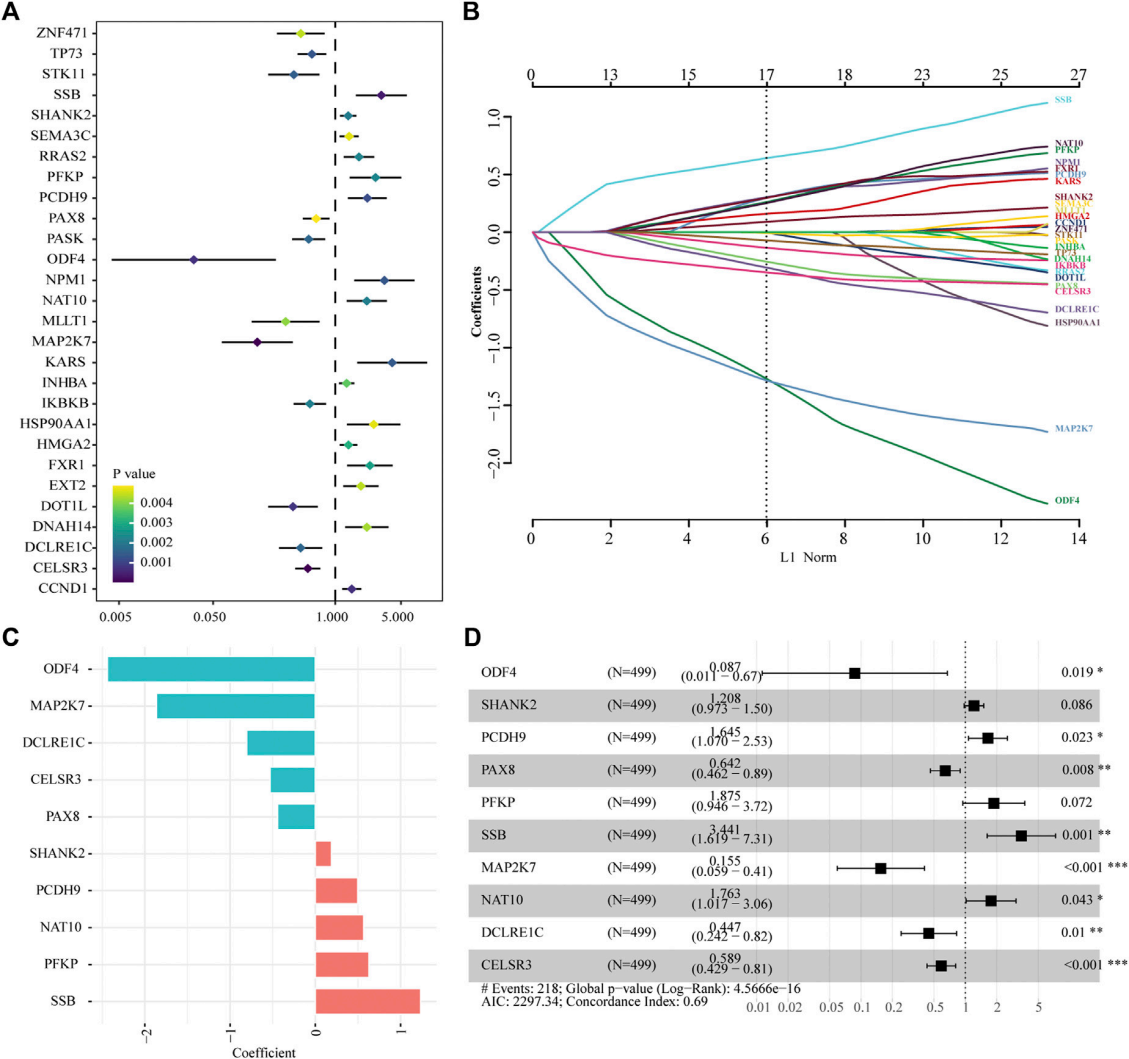
Identification of tumor driver genes in TCGA cohort. **(A)** Univariate Cox regression analysis to identify prognostic genes. **(B)** LASSO regression analysis to eliminate collinearity. **(C)** Coefficients of the 10 genes included in the signature. **(D)** Multivariable Cox proportional-hazards regression analysis of the 10 prognostic genes.

A total of 616 overlapping tumor-driver genes were acquired between TCGA and GSE41613 gene expression profiles. The univariate Cox regression analysis conducted between the 616 genes and OS illustrated that 28 genes were significantly correlated with OS (*p* < 0.005; Figure 2A). To identify the best prognostic genes and build a simplified prognostic model, we applied the LASSO Cox regression algorithm to the 28 prognosis-related genes. Eleven genes were excluded (Figure 2B). The 17 genes that met the screening criteria were subjected to the multivariate Cox regression analysis, at which point an optimal prognostic model was created (Figures 2C,D). The risk score was calculated as follows: risk score = (1.236 * SSB) + (0.628 * PFKP) + (0.567 * NAT10) + (0.498 * PCDH9) + (0.189 * SHANK2) + (−0.443 * PAX8) + (−0.529 * CELSR3) + (−0.806 * DCLRE1C) + (−1.865 * MAP2K7) + (−2.439 * ODF4). The risk score was computed for every case in the two cohorts, and all cases were classified into low- and high-risk groups based on the median threshold.

### Confirmation and validation of the gene signature

**FIGURE 3:**
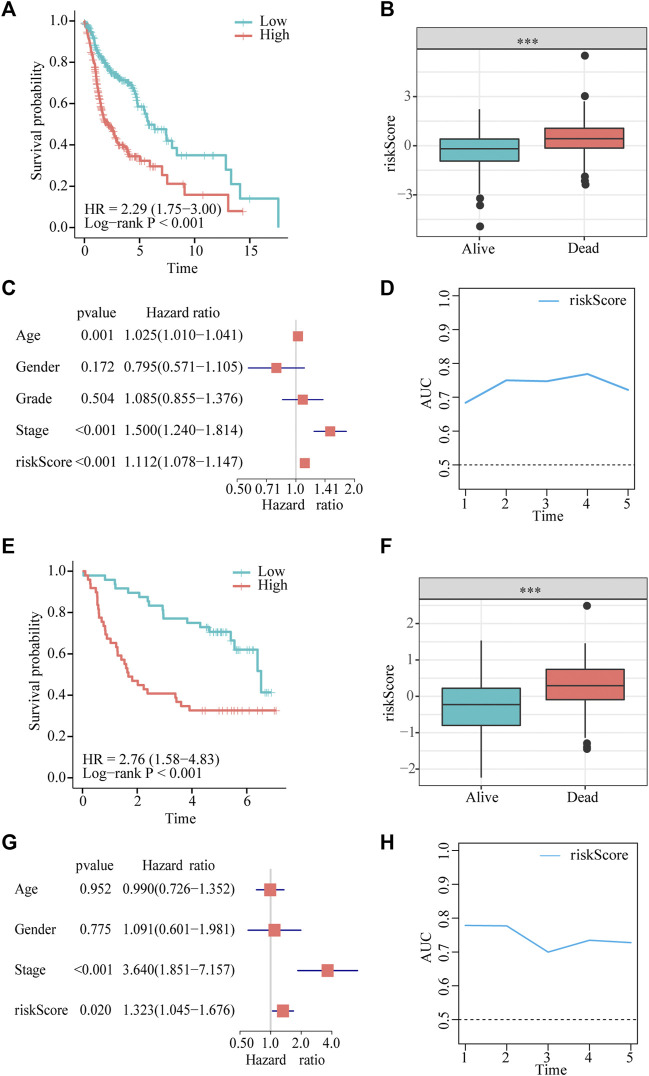
Gene signature predicts overall survival (OS) in TCGA and GEO datasets. **(A,E)** Kaplan-Meier analysis shows that HNSCC patients with higher risk scores have a poor prognosis. **(B,F)** The risk score is significantly elevated in patients with HNSCC who died during the follow-up. **(C,G)** Multivariate Cox regression analysis shows that the risk score is an independent prognostic factor for OS. **(D,H)** tROC analysis shows that the risk score is an accurate variable for survival prediction.

Patients in the two datasets were divided into low- and high-risk groups using the median risk score. Kaplan–Meier curves revealed that patients with HNSCC in the high-risk group had significantly shorter survival times than those in the low-risk group from TCGA cohort (hazard ratio [HR] = 2.29, *p* < 0.001, Figure 3A). Risk scores were significantly higher in patients who died than in those who were alive (Figure 3B). The multivariate Cox regression analysis showed that the age, stage, and risk score in TCGA dataset were independent predictors of survival outcomes in patients with HNSCC (Figure 3C).In addition, the tROC analysis showed strong performance of our prognostic model, with mean AUC values above 0.7 (Figure 3D).

To demonstrate the robustness of the prognostic model, we validated its performance using the *GSE41613* dataset. The results of the Kaplan–Meier analysis indicated that OS was lower in the high-risk group than in the low-risk group (HR = 3.11, *p* < 0.001, Figure 3E). The risk score was significantly higher in patients who died than in those who were alive (Figure 3F). Consistent with the results of multivariate Cox regression in TCGA cohort, the risk score was also an independent prognostic factor for patients with HNSCC with OS (HR = 1.323, *p* = 0.02, Figure 3G). tROC results in the validation dataset showed very high accuracy of the prognostic model, particularly in predicting 1- and 2-year survival, with AUC values close to 0.8 (Figure 3H).

**FIGURE 4:**
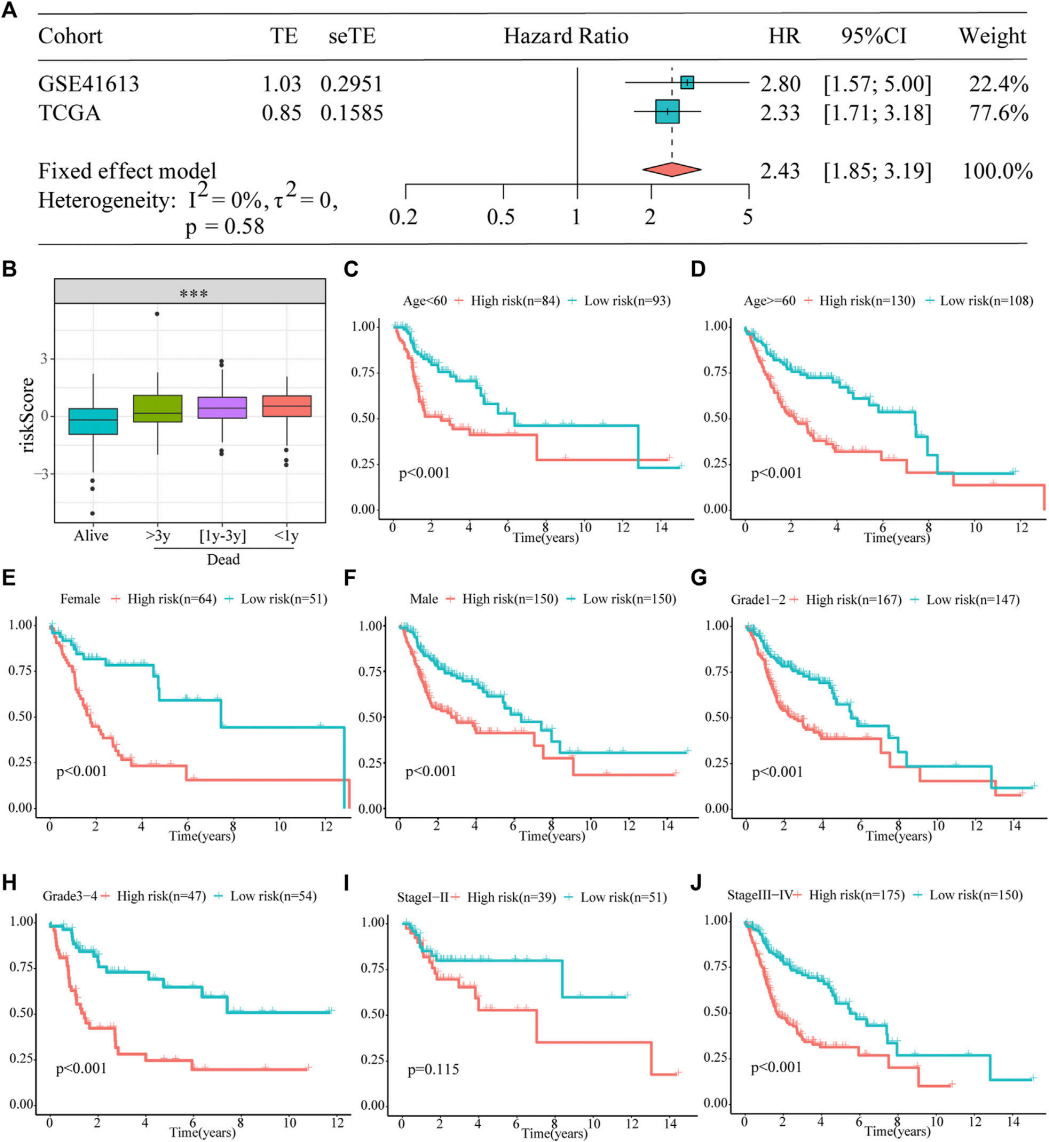
Prognostic signature can be used as a marker of survival in pooled cohorts and subgroups. **(A)** Meta-analysis. **(B)** The risk score is higher for patients who died than for those who were alive, particularly in the group with shorter survival. **(C–J)** Kaplan–Meier survival analysis of the signature between the high- and low-risk groups in TCGA cohort stratified by different clinicopathological parameters.

Furthermore, a meta-analysis was conducted to analyze the prognostic value of the prognostic models developed in the combined cohort. It showed that the prognosis of patients in the high-risk subgroup was worse than that of those in the low-risk subgroup (pooled HR = 2.43; 95% confidence interval: 1.85–3.19; Figure 4A). The association between survival time and the risk score was further investigated in TCGA dataset, and patients who died had higher risk scores. Particularly, those who survived for <1 year had the highest risk scores (Figure 4B). In addition, to explore the prognostic reliability and stability of the signature in different clinical groups, Kaplan–Meier analyses were used to plot survival curves and evaluate survival differences in the pooled cohort to visualize prognostic values. The curves indicated that patients in the high-risk group presented a drastically increased risk of dying among some patients with HNSCC, including age (<60 years, ≥ 60 years; Figures 4C,D), ex (female, male; Figures 4E,F), grade (1–2, 3–4; Figures 4G,H), and stage (I–II, III–IV; Figures 4I,J), demonstrating that the signature was a sturdy prognostic biomarker.

### Genetic alteration of 10 genes in HNSCC

**FIGURE 5:**
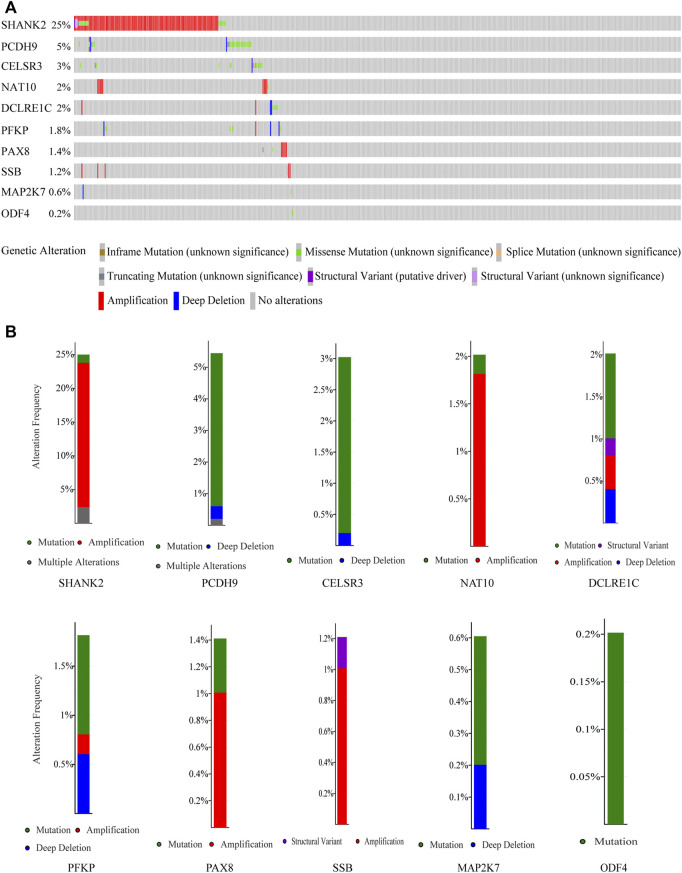
Genetic alterations of the 10 genes analyzed utilizing cBioPortal. **(A)** Genetic alterations in each of the 10 genes in the prognosis signature. **(B)** Specific alteration frequency of the 10 genes with the study of clinical samples.

By analyzing HNSCC samples from the cBioPortal database, we assessed alterations in the 10 genes used to create the prognostic signature. The total mutation frequency was 42.2%, and all genes in the prognostic signature had amplified or missense mutations in patients with HNSCC, suggesting that these 10 genes play an important role in HNSCC (Figure 5A). Among the 496 patients with HNSCC, 1.21% had mutations, 21.37% had amplifications, and 2.42% had multiple alterations in *SHANK2*; 4.84% had mutations, 0.2% had multiple alterations, and 0.4% had deep deletions in *PCDH9*; 2.82% had mutations, 0.2% had deep deletions in *CELSR3*, 0.2% had mutations, and 1.81% had amplifications in *NAT10*; 1.01% had mutations, 0.2% had structural variants, 0.4% had amplifications, and 0.4% had deep deletions in *DCLRE1C*; 1.01% had mutations, 0.2% had amplifications, and 0.6% had deep deletions in *PFKP*; 1.01% had amplifications and 0.2% had structural variants in *SSB*; 0.4% had mutations and 0.2% had deep deletions in *MAP2K7*; and 0.2% had mutations in *ODF4* (Figure 5B).

### Identification and validation of diagnostic feature biomarkers

**FIGURE 6:**
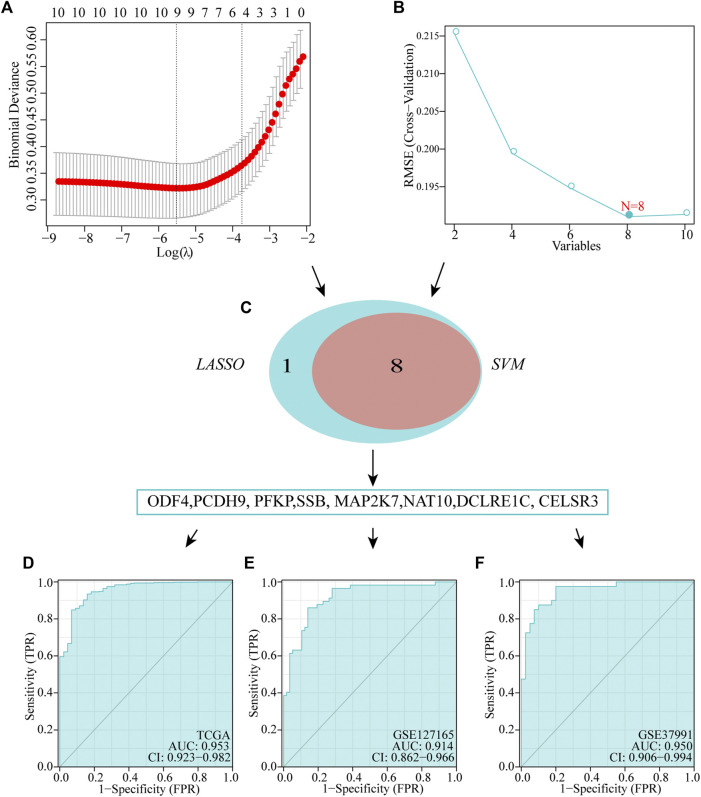
Establishment of multigene diagnostic signature. A total of eight genes have been selected through LASSO **(A)** and SVM **(B)** analyses, with AUCs of 0.953 **(D)**: TCGA, 0.978 **(E)**: GSE127165, and 0.950 **(F)**: GSE37991.

We executed two different algorithms, namely SVM and LASSO, on the 10 genes used to construct the prognostic model to improve the accuracy of the diagnostic model. Ten genes were screened using the LASSO algorithm, and nine candidate genes were obtained (Figure 6A). Screening was performed using the SVM-RFE algorithm, and eight genes were identified (Figure 6B). Subsequently, by taking the intersection of the genes obtained using the two algorithms, we obtained eight genes to construct diagnostic markers (Figure 6C). Finally, a multivariate logistic regression analysis was used to construct a diagnostic model based on these eight genes. The model formula is as follows: -8.3049 + (−9.9087*ODF4) + (−2.3662*PCDH9) + (0.8175*PFKP) + (1.2565*SSB) + (0.2716*MAP2K7) + (0.5908*NAT10) + (−1.769*DCLRE1C) + (7.2511*CELSR3). In TCGA dataset, ROC showed that our diagnostic model had a high AUC value (0.953), indicating that the model could accurately differentiate between HNSCC and normal tissues (Figure 6D). In addition, we evaluated the performance of the diagnostic model using two external datasets and found that the model had a high diagnostic value (*GSE127165*: AUC = 0.914; *GSE37991*: AUC = 0.950; Figures 6E,F).

### Sensitivity of patients with HNSCC in high- and low-risk groups to different chemotherapeutic drugs

**FIGURE 7:**
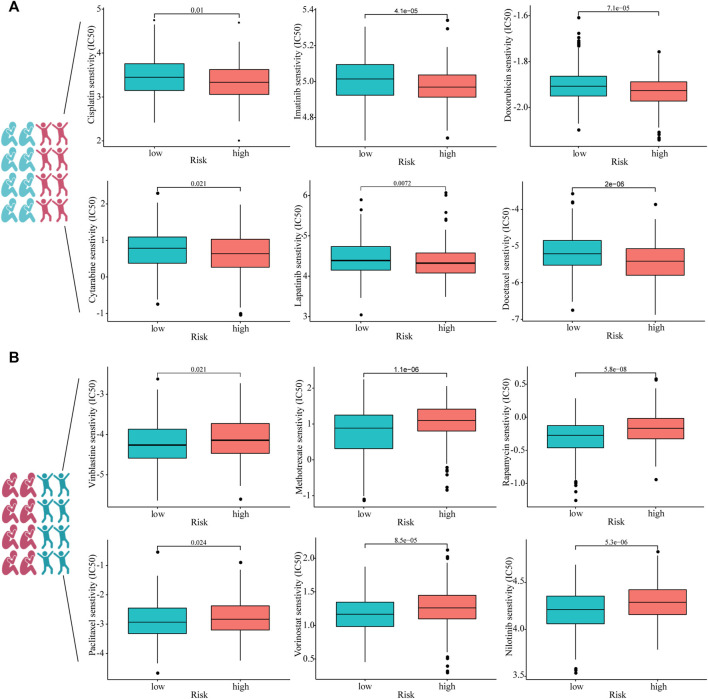
Estimated drug sensitivity in patients with HNSCC in high- (*n* = 249), **(A)** and low-risk (*n* = 250), **(B)** groups.

To investigate whether or not prognostic models can guide the use of chemotherapeutic drugs in clinical practice, we compared IC50 of the high- and low-risk groups with those of various common chemotherapeutic drugs and found higher sensitivity to cisplatin, imatinib, doxorubicin, cytarabine, lapatinib, and docetaxel in the high-risk group (Figure 7A). In addition, IC50 of vinblastine, methotrexate, rapamycin, paclitaxel, vorinostat, and nilotinib was lower in patients with lower risk scores, suggesting that patients with HNSCC in the lower-risk group were sensitive to these drugs (Figure 7B).

### Downregulated expression of *DCLRE1C* in HNSCC

**FIGURE 8:**
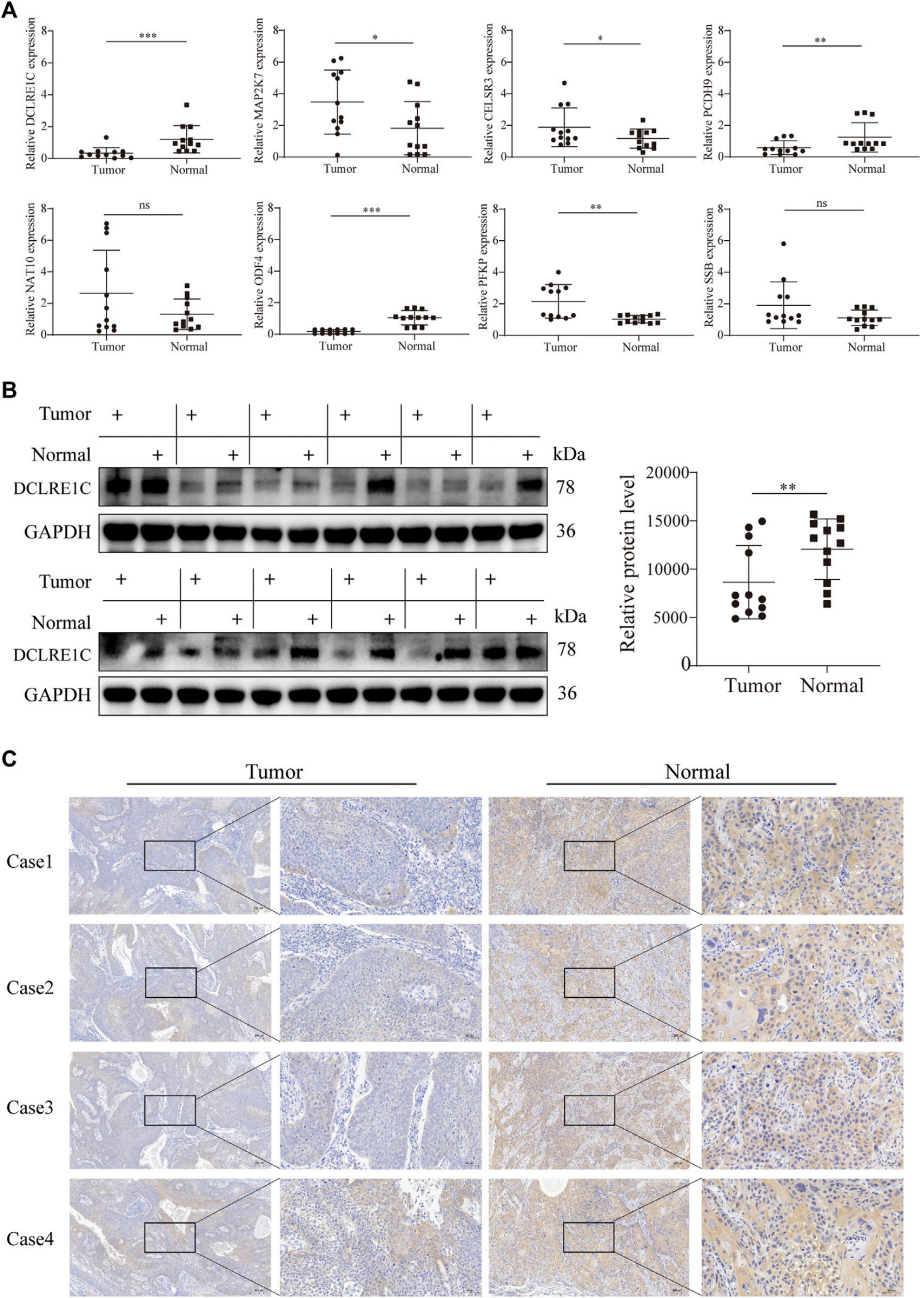
Detection of gene expression in tissue samples using qRT-PCR, WB and IHC.**(A)** qRT-PCR analysis of mRNA levels in tumor and adjacent normal tissues.**(B,C)** Western blotting, immunohistochemistry analysis of DCLRE1C protein levels in tumor and adjacent normal tissues.

To detect differences in mRNA expression of the eight signature genes, we conducted reverse transcription qRT-PCR to detect 12 pairs of cancer and normal tissues (Figure 8A). The mRNA expression levels of *DCLRE1C*, *PCDH9*, and *ODF4* in tumors were significantly lower than those in the normal tissues. In contrast, *MAP2K7*, *CELSR3*, and *PFKP* were overexpressed in tumor tissues. However, mRNA expression levels of *SSB* and *NAT10* did not differ between HNSCC and adjacent normal tissues (Figure 8A). Furthermore, Western blotting and immunohistochemistry analyses revealed that *DCLRE1C* was expressed at substantially lower levels in HNSCC tissues than in corresponding normal tissues (Figures 8B,C).

## Discussion

HNSCC is a common cancer worldwide. Even after new therapeutic methods and significant advances in clinical research, the long-term life expectancy and survival rate of patients with HNSCC remain unsatisfactory because of tumor recurrence or metastasis. Although clinical patients have the same medical and pathological levels, because of the complicated etiological elements and high heterogeneity, predicting the survival rate of patients with HNSCC is difficult.

Currently, effective biomarkers with high accuracy for the diagnosis and prognosis of HNSCC are lacking (Zhao et al., 2010). Therefore, establishing accurate prognostic models and diagnostic markers is necessary. However, bioinformatics research has often focused on a single database or only on the prognostic value, which has some limitations (Cui et al., 2020). In recent years, with the rapid development of bioinformatics, numerous novel gene biomarkers have been discovered as critical modulators for the diagnosis and survival prognosis prediction of various diseases (Wang et al., 2009; Zhao and Bai, 2020). Future research should identify clinically significant genes, predict their functions, and explore their prognostic value using bioinformatics.

Altered expression levels of tumor-driver genes may promote malignant biological behavior of tumor cells (Bossi et al., 2016; Zhao et al., 2019). Based on 2,372 tumor-driver genes, new models that can predict the prognosis of patients with HNSCC as well as accurate diagnostic markers may be identified through the combined use of bioinformatics approaches. In this study, we used WGCNA to identify modules associated with HNSCC and successively used univariate, LASSO, and multivariate Cox regression models to filter genes to establish a tumor driver-related gene signature. The performance of this signature was verified using the *GSE41613* cohort. In addition, although the meta-analysis and subgroup analysis were based on TCGA cohort, our prognostic model could discriminate between high- and low-risk populations. These results demonstrated the superior predictive performance of the proposed model. In clinical practice, this is conducive to prolonging patient survival if an accurate diagnosis can be made for patients with HNSCC. The diagnostic model that we constructed showed excellent performance in both the training and independent validation sets. However, the constituent genes of the two models are not identical. There may be the following reasons. First, models were built on different people. In more detail, prognostic model were constructed for tumor patients, while diagnostic model were constructed based on normal cohorts and tumor patients. Second, the algorithms used in the construction of the two models were different. The data types in the prognostic model were survival data and in the diagnostic model were dichotomous variables, which were analysed using LASSO “Cox” and “binomial” regressions respectively using the glmnet R package. Third, the diagnostic model use the SVM-RFE algorithm, which is based on the SVM interval maximisation principle and uses the support vector machine weight coefficients as a scoring criterion to rank genes, eliminating one gene at a time with the lowest ranking score, until the final set of ranked features is obtained. After ten genes were screened by this step, SHANK2 and PAX8 genes were filtered out and were not involved in the construction of the diagnostic model. In addition, SHANK2 and DCLRE1C of these ten genes were not differentially expressed in normal and tumor tissues. A number of previous studies have performed differential expression analyses prior to constructing prognostic models (Long et al., 2020; Yan et al., 2022). However, in this study we were concerned that prognosis-related genes would be filtered out during differential analysis, so we included all genes directly in the analysis. This is similar to previous studies (Cai et al., 2021). Regardless, our findings suggest that the performance of these two models is very strong. If the model can be applied to clinical work in the future, this will surely reduce the public health burden.

To date, chemotherapy remains an effective treatment option for patients with HNSCC, and selection of the exact chemotherapeutic drug can reduce patient suffering and prolong life expectancy. Therefore, we maximized the benefit rate for patients by predicting the sensitivity of chemotherapeutic drugs in different subgroups of the population.

In this study, qRT-PCR showed that the expression levels of two genes, *DCLRE1C* and *ODF4*, differed significantly between normal and tumor tissues. In addition, *ODF4* was expressed at a lower level in the tumor group than in the *DCLRE1C* group. Therefore, we selected *DCLRE1C* to continue the subsequent Western blotting and immunohistochemistry analyses, which were consistent with the qRT-PCR results.

Some genes used to build prognostic models have been studied in other cancers. For example, *PFKP* expression levels are negatively correlated with the prognosis of lung cancer, and the proliferation rate of cells with low *PFKP* expression is significantly reduced (Shen et al., 2020). In addition, *PFKP* can be used as a therapeutic target in patients with breast cancer (Yeerken et al., 2020). In oral cancer, regulation of *PFKP* expression promotes cell proliferation, migration, and invasion (Chen et al., 2018). Recently, Zhang et al. demonstrated through a series of *in vivo* and *in vitro* experiments that reduced levels of *NAT10* expression could inhibit gastric cancer metastasis (Zhang et al., 2021). In a study on hepatocellular carcinoma, *NAT10* expression was significantly upregulated in cancerous tissues, and its expression level was negatively correlated with the OS of patients, suggesting that it is an oncogene (Li et al., 2017). However, in our study, reverse transcription qRT-PCR showed that the difference in expression levels of *NAT10* in cancer and normal tissues was not significant. The reason for this result may be the insufficient sample size and the fact that tumor subtypes are not highly representative. Decreased *PCDH9* expression is associated with the metastasis of gastric cancer cells (Chen et al., 2015). High levels of miR-200a-3p promote the proliferation of ovarian cancer cells by targeting *PCDH9* (Shi et al., 2019). These results indicated that *PCDH9* is a tumor-suppressor gene with low expression in tumor tissues, consistent with our reverse transcription qRT-PCR results. *PAX8* promotes the proliferation of gastric cancer cells (Bie et al., 2019). Low expression of *CELSR3* significantly reduces the migration and invasion of lung adenocarcinoma cells (Li et al., 2021). *CELSR3* expression may serve as a prognostic biomarker in patients with prostate cancer and may predict poor outcomes (Chen et al., 2021). In addition, *CELSR3* is highly expressed in liver and oral cancers and associated with a poor prognosis (Gu et al., 2019; Zheng et al., 2022). In another study, *ODF4* was significantly highly expressed in breast cancer tissue and could be used in combination with other biomarkers to differentiate between cancer and normal tissues (Kazemi-Oula et al., 2015). This is in contrast with the results of our reverse transcription qRT-PCR analyses, and it could play different biological roles in different cancers. Taken together, the potential mechanisms of these genes in HNSCC require further detailed investigations.

The present study has some limitations. First, the sample size from our center was small, and more samples with detailed clinicopathological and prognostic information are necessary to further investigate the performance of the risk signature in predicting HNSCC progression and prognosis. Second, we require a longer follow-up period to further compare HNSCC between low- and high-risk groups because of the relatively limited follow-up period. In addition, functions of the eight genes involved in the malignant progression of HNSCC were not investigated. Further research is required to explore the mechanisms of the eight genes involved in tumor progression.

## Conclusion

We developed a new 10-gene model using a combination of bioinformatics approaches, which could accurately predict the prognosis of HNSCC and identify the chemotherapeutic drugs from which patients benefit, thus facilitating personalized management of patients with HNSCC. In addition, we successfully constructed a biomarker capable of accurately diagnosing HNSCC, which can be used in the molecular diagnosis of HNSCC.

## Data Availability

The datasets presented in this study can be found in online repositories. The names of the repository/repositories and accession number(s) can be found below: https://www.ncbi.nlm.nih.gov/geo/, GSE41613 https://www.ncbi.nlm.nih.gov/geo/, GSE127165.
