# Supplementary material for: Identification and validation of a novel tumor driver gene signature for diagnosis and prognosis of head and neck squamous cell carcinoma
Source: Front Mol Biosci. 2022 Oct 20;9:912620. doi: 10.3389/fmolb.2022.912620 (PMC9631213; doi:10.3389/fmolb.2022.912620)
Supplement: Supplementary file 1 [file Table1.docx]

Table S1. Primer sequences for qRT-PCR.

| **Primer name** | **GC content (%)** | **Tm (°C)** | **Annealing Temperature (°C)** | **Primer sequence(5′-3′)** |  |
| --- | --- | --- | --- | --- | --- |
|  |  |  |  |  |  |
| DCLRE1C FORWARD | 45.5 | 54.02 | 60 | TGA GGA CTG GAG AGA GTT CAT A |  |
| DCLRE1C REVERSE | 40.9 | 52.91 |  | GAC ATT TGG ATA TGC GTT CAC A |  |
| MAP2K7 FORWARD | 50 | 54.65 | 60 | AGA TCA AGC TCT GCG ACT TC |  |
| MAP2K7 REVERSE | 45.5 | 53.67 |  | CTT GCA GTT CTT GTA GGG AAA C |  |
| CELSR3 FORWARD | 45.5 | 45.5 | 60 | TTA GCT CTT TGT CCG GAG TCT T |  |
| CELSR3 REVERSE | 50 | 57.06 |  | TTC AAT CCC CAG CTC CTC ATT C |  |
| PCDH9 FORWARD | 40.9 | 53.08 | 60 | CCA ACA ACA TTC AAG CCT AAC A |  |
| PCDH9 REVERSE | 45.5 | 53.64 |  | GTT TAG AAA GGG TGT CAC AAC C |  |
| NAT10 FORWARD | 45.5 | 53.76 | 60 | CAC GGA ATA TGG TGG ACT ATC A |  |
| NAT10 REVERSE | 45.5 | 53.96 |  | GTT GAA AAG TCC CAT CAA CTG G |  |
| ODF4 FORWARD | 52 | 59.99 | 60 | GCA CAG TCA CCT TCA TCT TCT CCA C |  |
| ODF4 REVERSE | 50 | 59.97 |  | AGG ATA AGC ACC AGC CAA CCA ATG |  |
| PFKP FORWARD | 50 | 54.29 | 60 | GTA CTT CAT CTA CGA GGG CTA C |  |
| PFKP REVERSE | 47.6 | 54.89 |  | GATC ACA CAC AGG TTG GTG AT |  |
| SSB FORWARD | 40.9 | 53.41 | 60 | ACG GGA CAA GTT TCT AAA GGA A |  |
| SSB REVERSE | 45.5 | 53.53 |  | GTC TGT TGT TAG ACG GTT CAA C |  |
| GAPDH FORWARD | 50 | 55 | 60 | GTA TCG TGG AAG GAC TCA TGA C |  |
| GAPDH REVERSE | 40.9 | 53.36 |  | ACC ACC TTC TTG ATG TCA TCA T |  |
