# Supplementary material for: Identification and validation of a novel tumor driver gene signature for diagnosis and prognosis of head and neck squamous cell carcinoma
Source: Front Mol Biosci. 2022 Oct 20;9:912620. doi: 10.3389/fmolb.2022.912620 (PMC9631213; doi:10.3389/fmolb.2022.912620)
Supplement: Supplementary file 2 [file Table2.docx]

Table S2. List of genes with correlations greater than 0.4 for the three modules (red, blue and black).

|  |  |  |  |  |  |  |
| --- | --- | --- | --- | --- | --- | --- |
| MET | AJAP1 | PNPLA7 | PMS1 | PLCH2 | POLRMT | KPNB1 |
| TNC | TLL1 | RYR1 | MSH2 | MRPS31 | NUP93 | DNMT3B |
| PTPRK | MARK3 | ASB15 | MAX | ACD | NUP133 | AURKA |
| CCND1 | DISP2 | GDF5 | CDK4 | WDR12 | LEMD2 | HDAC2 |
| ELF3 | SDK2 | PRKCQ | ATR | OXA1L | IRF2 | CC2D1A |
| ACVR1 | LFNG | OBSCN | CAMTA1 | TMEM216 | GRIN2D | SMU1 |
| SOX9 | DLL1 | ADCY1 | CNOT3 | CNOT9 | DAZAP1 | DENND4B |
| CSMD3 | GOT1 | SPHKAP | SOCS1 | ADGRV1 | CBWD3 | CAMKK2 |
| RET | ATP5F1B | XPNPEP2 | DDX10 | DOT1L | BRD7 | SPTBN5 |
| MYH9 | SHANK2 | APOBEC2 | SFPQ | DDX51 | BCL2L11 | RBM39 |
| MSN | PIK3R1 | CDH19 | MDM2 | KARS | ATXN3 | SERHL2 |
| FLNA | RAC1 | LIMCH1 | CCNE1 | FANCM | ZNF133 | AFMID |
| TEC | MYOD1 | ADH1B | DDX5 | PASK | TTK | TAF11 |
| SPECC1 | IDH2 | PEG3 | KLF6 | SERPINA12 | SPRR2B | KALRN |
| EXT2 | KIT | XIRP2 | FEN1 | SKP2 | METTL3 | PERM1 |
| CDK6 | NTRK3 | AOX1 | PALB2 | MAP3K21 | ZNF572 | SIN3B |
| SIRPA | ERBB4 | TYRP1 | BUB1B | IMP4 | NUDT11 | ERF |
| SDC4 | CLTCL1 | LBP | BRIP1 | LRRC7 | NETO2 | B4GALT3 |
| RAP1GDS1 | AR | ADRA1A | SUZ12 | TFDP1 | HMGB1 | STRADA |
| PPFIBP1 | FAM135B | ESRRG | EED | MAGEA1 | GRB2 | STK11IP |
| XPA | MITF | ZNF677 | TFG | GPS2 | DUSP9 | SMG5 |
| MYO5A | ZBTB16 | MLXIPL | TFRC | PFKP | AKAP8 | SMC4 |
| NACA | SH3GL1 | AGTR2 | TAF15 | DNAH14 | ANKLE2 | SMC2 |
| NDRG1 | RSPO3 | CTNNA3 | TCEA1 | CHD5 | RASGEF1A | NCAPH2 |
| MN1 | PAX7 | IGFN1 | TCF3 | SLCO1B1 | ZWILCH | NCAPG2 |
| MAF | ZNF429 | SPTB | SRSF3 | G3BP1 | PDIA4 | NCAPD2 |
| LASP1 | MYH11 | PRX | SS18 | SNRPA | MKRN3 | MMS22L |
| ITGAV | LMO1 | PLCE1 | SS18L1 | FCER1A | CDC25C | IWS1 |
| IGF2BP2 | CTNND2 | LRIG1 | SET | MCMBP | MNAT1 | FOXM1 |
| HOXC11 | BMP5 | CARNS1 | SDHA | SSB | NEK2 | FOXK2 |
| HOXC13 | NFIB | BOC | RFWD3 | TCERG1 | ESPL1 | EIF2AK1 |
| HMGA2 | TPM3 | UACA | RMI2 | HORMAD1 | SHKBP1 | E2F3 |
| CHST11 | ANK1 | ODAM | RAD17 | PAIP1 | MST1R | DCLRE1C |
| NRG1 | C1orf127 | NTN4 | POLD1 | CDKN2B | ADGRL1 | CELSR3 |
| FKBP9 | PRG4 | MYOCD | POU5F1 | HINFP | ABCC5 | VARS2 |
| EXT1 | ABCC9 | MPO | NONO | KLHDC4 | SAGE1 | SLC4A5 |
| ABL2 | CALCR | CAP2 | ZCCHC8 | BICRA | WDR75 | RAB40A |
| SEMA3C | ZNF844 | BHMT2 | YWHAE | CNTN5 | CEP83 | PDCD2L |
| DRD5 | RCAN2 | ZNF471 | NAB2 | ZAN | TYSND1 | PDAP1 |
| MMP13 | PRUNE2 | MEF2D | NCKIPSD | ZNF638 | PDCD6 | OMA1 |
| ODF4 | ADAMTSL3 | PRB2 | MTCP1 | CDH24 | WDR24 | MORC4 |
| HDAC9 | TTN | MEN1 | MDM4 | CD58 | U2AF2 | INTS12 |
| AJUBA | NMRK2 | DAXX | MLF1 | RIMS2 | SRSF1 | EIF2S2 |
| MYO1B | CCDC63 | ASXL1 | MLLT1 | ZIC4 | NLN | CEP76 |
| OR2L13 | SLC38A3 | CDKN2A | USP6 | PNLIPRP3 | RTTN | ALKBH6 |
| SORCS2 | SCN5A | CHEK2 | HSP90AA1 | ZSWIM3 | FAM111B | TPX2 |
| CYP2C9 | PPP1R3A | PIK3CA | HSP90AB1 | CCNL1 | FLYWCH1 | ZNF180 |
| PTPN12 | NRG2 | FUBP1 | HOXA13 | DBR1 | SAMM50 | ZRANB3 |
| RHOB | MYH2 | PPP6C | HOXA9 | NEURL4 | ABCF2 | TFAP4 |
| INHBA | ADGRD1 | KNSTRN | HMGA1 | MYBL1 | TMEM201 | LAPTM4B |
| SLC6A15 | F8 | U2AF1 | TRIM27 | FXR1 | USP5 | FMR1 |
| GLI3 | CACNA2D1 | BAP1 | GMPS | TMEM199 | MAP2K7 | ZIC1 |
| RHBG | ADGRB3 | SMARCA4 | FUS | HRNR | SRRT | PTPN2 |
| LAMA3 | ABLIM2 | KEAP1 | ERCC3 | ATAD2 | NKTR | UBA2 |
| PIK3CD | SLITRK5 | STK11 | ETV4 | LRIG2 | NCAPD3 | HOXA10 |
| NOXA1 | TPO | RBM10 | ETV5 | NADK | PABPC1L |  |
| TRIO | ADGRA1 | CALR | FGFR1OP | RBMX | NAT10 |  |
| PLEC | DCLK1 | EZH2 | FANCE | DCTD | MYBL2 |  |
| AHNAK2 | PCDH9 | BRCA1 | FANCG | MDC1 | C6orf136 |  |
| CHPF2 | MYO18B | BRCA2 | CYP2C8 | TRIM37 | DNAAF5 |  |
| GNB1 | DMD | SMARCD1 | DDB2 | TMPRSS13 | ADD2 |  |
| NAV1 | AQP12A | TERT | DDIT3 | ENTPD6 | TWNK |  |
| NAV3 | ACTG1 | SMARCB1 | DEK | NOP58 | KHSRP |  |
| RRAS2 | KCNN3 | RNF43 | DGCR8 | TBP | SLURP1 |  |
| IRS1 | MAMSTR | FH | DROSHA | MAPKAPK5 | PPP6R2 |  |
| CSPG4 | USP13 | MSH6 | EIF3E | C2CD6 | SALL3 |  |
| COL7A1 | KCNA4 | CASP8 | EIF4A2 | NFKBIA | CYP4F22 |  |
| OSMR | MYH7 | SPOP | CNTRL | MLH3 | UBE2A |  |
| ERRFI1 | NEB | ERCC2 | CLP1 | MSH5 | ZNF830 |  |
| RTL9 | HSPB8 | TSC1 | CHIC2 | TEX15 | PGAP2 |  |
| SLC6A2 | DLK1 | FGFR4 | CEP89 | GPRIN1 | CLK3 |  |
| ALOX12 | TGFBR3 | NPM1 | CHCHD7 | HAVCR1 | TUBGCP6 |  |
| CARD10 | DYSF | BTG1 | CCNB1IP1 | MRPL18 | EPHB4 |  |
| IL1B | EYA4 | XPO1 | CCNC | EIF4A1 | CCNB2 |  |
| SNX7 | CRB1 | POT1 | CARS | DDX55 | PNN |  |
| ARPC1B | IGSF10 | MAP3K13 | KNL1 | HNRNPU | METTL14 |  |
| SOAT1 | ZBTB20 | TBL1XR1 | CASP3 | VMA21 | HNRNPK |  |
| ELAVL2 | PGR | CBFB | BLM | TRAF2 | C2CD4C |  |
| SLITRK6 | PKHD1L1 | FANCA | STIL | NXF1 | ECT2 |  |
| ACTB | MAFA | MUTYH | RPN1 | THAP12 | HSF1 |  |
| MAGEA6 | PPP1R9A | RECQL4 | RBM15 | H2AFV | PRKCI |  |
| JAG1 | KCTD8 | AXIN1 | PRCC | C6orf48 | YEATS4 |  |
| FGF5 | MEF2C | DNMT1 | PAX8 | MKI67 | GPATCH4 |  |
| TENM2 | LRRTM1 | GNA11 | HOXD13 | SMC3 | GPAT4 |  |
| PDHB | PROKR2 | EIF1AX | HOXD11 | KDM1A | MAP2K3 |  |
| GNA12 | FBXO31 | HNRNPA2B1 | HOXA11 | CENPF | NDC80 |  |
| DSE | CD36 | IKBKB | FIP1L1 | TLE1 | NLRP1 |  |
| C15orf48 | MYOM2 | FLCN | FANCD2 | UCHL5 | ZDHHC4 |  |
| FHOD1 | MAS1L | MLH1 | FANCC | PLCD1 | RNF168 |  |
| KRT13 | PRKAB2 | PIM1 | CRNKL1 | CEP152 | CREBZF |  |
| TGFA | FBXO32 | EWSR1 | CNBP | ADCK1 | PPM1J |  |
| ATP10D | ANK2 | ZRSR2 | WDCP | FLG | RIOK2 |  |
| GSDME | MYH4 | SRSF2 | BCL2L12 | ZIC3 | CNTLN |  |
| NCF2 | MYOM3 | RAD51B | BARD1 | BRD8 | CENPJ |  |
| PTGFRN | SORCS1 | SYK | ATIC | TGIF1 | CPNE1 |  |
| CDH13 | CLCN4 | NSD2 | APOBEC3B | SF1 | FAM72D |  |
| TGFBR1 | AKAP6 | RAD21 | MST1 | RHEB | IQCG |  |
| PHLDA1 | KCNAB1 | PSIP1 | ANKRD36 | RFC1 | PFAS |  |
| PORCN | DTNA | POLQ | CD3EAP | PTMA | TP73 |  |
| CDHR1 | COL25A1 | POLE | LCE4A | PTPDC1 | ASPM |  |
